# Supplementary material for: Evolution of herbivore-induced early defense signaling was shaped by genome-wide duplications in Nicotiana
Source: eLife. 2016 Nov 4;5:e19531. doi: 10.7554/eLife.19531 (PMC5115867; doi:10.7554/eLife.19531)
Supplement: Supplementary file 1. — (A) Genes induced by FAC in all six Nicotiana species. Green and red colors indicate e3 ubiquitin-protein ligase and transcription factors, respectively. (B) Genes which showed lower expression in samples induced by M. sexta OS than by FAC in both N. attenuata and N. pauciflora. Numbers in each column indicate the TMM-normalized FPKM values. (C) Summary of RNA-seq reads from six closely related Nicotiana species. (D) Primers used for qPCR and VIGS in this study. DOI: http://dx.doi.org/10.7554/eLife.19531.020 [file elife-19531-supp1.docx]

**Supplementary file 1A. Genes induced by FAC in all six *Nicotiana* species.** Green and red colors indicate e3 ubiquitin-protein ligase and transcription factors, respectively.

| **Gene ID** | **Annotation** | **Note** | **Best Blast Hit** |
| --- | --- | --- | --- |
| NIATv7_g05178 | e3 ubiquitin-protein ligase pub23-like | e3 ubiquitin-protein ligase | XP_004228871 |
| NIATv7_g34426 | e3 ubiquitin-protein ligase pub23-like | e3 ubiquitin-protein ligase | XP_004241334 |
| NIATv7_g16579 | ap2 erf domain-containing transcription factor | Transcription Factor | XP_004237817 |
| NIATv7_g30738 | ap2 erf domain-containing transcription factor | Transcription Factor | XP_004237144 |
| NIATv7_g12925 | heat stress transcription factor a-4a-like | Transcription Factor | XP_004243245 |
| NIATv7_g36386 | transcription factor tt2-like | Transcription Factor | XP_004236420 |
| NIATv7_g09195 | PLATZ transcription factor family | Transcription Factor | XP_009790819 |
| NIATv7_g12331 | probable wrky transcription factor 28-like | Transcription Factor | XP_004233015 |
| NIATv7_g35285 | probable wrky transcription factor 41-like | Transcription Factor | XP_004234275 |
| NIATv7_g27487 | probable WRKY transcription factor 48 | Transcription Factor | XP_009770697 |
| NIATv7_g35699 | endochitinase pr4 |  | XP_004237833 |
| NIATv7_g00113 | auxin-regulated protein |  | NP_001234635 |
| NIATv7_g01329 | PREDICTED: uncharacterized protein LOC101261228 |  | XP_004238950 |
| NIATv7_g01695 | ring-h2 finger protein atl3-like |  | XP_004235522 |
| NIATv7_g04111 | vq motif-containing protein |  | XP_004237898 |
| NIATv7_g05562 | u-box domain-containing protein 21-like |  | XP_004250456 |
| NIATv7_g08158 | f-box protein skip27-like |  | XP_004233817 |
| NIATv7_g13317 | uncharacterized loc101205876 |  | XP_004232247 |
| NIATv7_g14560 | sugar transport protein 13-like |  | NP_001234785 |
| NIATv7_g14832 | isoflavone 2 -hydroxylase-like |  | XP_004238116 |
| NIATv7_g15963 | phospholipase a1- chloroplastic-like |  | XP_004244860 |
| NIATv7_g17751 | predicted protein |  | XP_002322915 |
| NIATv7_g17823 | probable indole-3-acetic acid-amido synthetase -like |  | XP_004230721 |
| NIATv7_g23001 | PREDICTED: uncharacterized protein LOC100854721 isoform 1 |  | XP_003633026 |
| NIATv7_g23271 | basic 7s globulin-like |  | NP_001234249 |
| NIATv7_g23435 | uncharacterized loc101216872 |  | XP_002536881 |
| NIATv7_g29183 | nac domain ipr003441 |  | XP_002328928 |
| NIATv7_g30658 | thioredoxin reductase 2-like |  | XP_004232594 |
| NIATv7_g31295 | mitochondrial chaperone bcs1-like |  | XP_004234999 |
| NIATv7_g40482 | 4-coumarate-- ligase 1-like |  | XP_004235870 |
| NIATv7_g18591 | LURP-one-related 14-like isoform X2 |  | XP_009763498 |
| NIATv7_g26461 | F-box PP2-B15-like |  | XP_009795812 |
| NIATv7_g28238 | exocyst complex component EXO70A1-like |  | XP_009798500 |
| NIATv7_g58651 | PREDICTED: uncharacterized protein LOC104242562 |  | XP_009795939 |
| NIATv7_g59916 | RING-H2 finger ATL3-like |  | XP_009788965 |

**Supplementary file 1B. Genes which showed lower expression in samples induced by *M. sexta* OS than by FAC in both *N. attenuata* and *N. pauciflora.*** Numbers in each column indicate the TMM-normalized FPKM values.

| **Species** | ***N. attenuata*** | | | | | | | | | | | | ***N. pauciflora*** | | | | | | | | | | | | **Annotation** |
| --- | --- | --- | --- | --- | --- | --- | --- | --- | --- | --- | --- | --- | --- | --- | --- | --- | --- | --- | --- | --- | --- | --- | --- | --- | --- |
| **Treatments** | Water | | | FAC | | | OS*_Ms_* | | | OS*_Sl_* | | | Water | | | FAC | | | OS*_Ms_* | | | O*_SI_* | | |  |
| **Replicates** | 1 | 2 | 3 | 1 | 2 | 3 | 1 | 2 | 3 | 1 | 2 | 3 | 1 | 2 | 3 | 1 | 2 | 3 | 1 | 2 | 3 | 1 | 2 | 3 |  |
| NIATv7_g02972 | 1 | 1 | 2 | 24 | 42 | 31 | 17 | 21 | 8 | 22 | 33 | 11 | 1 | 1 | 1 | 5 | 5 | 4 | 2 | 2 | 2 | 3 | 3 | 3 | g-type lectin s-receptor-like serine threonine-protein kinase |
| NIATv7_g09323 | 4 | 8 | 6 | 28 | 49 | 29 | 19 | 24 | 20 | 24 | 31 | 19 | 2 | 3 | 2 | 11 | 8 | 13 | 4 | 6 | 6 | 3 | 3 | 4 | ammonium transporter 1 member 1-like |
| NIATv7_g12469 | 10 | 11 | 13 | 24 | 28 | 22 | 12 | 16 | 9 | 19 | 21 | 10 | 10 | 10 | 10 | 19 | 18 | 13 | 10 | 8 | 11 | 16 | 15 | 13 | dis3-like exonuclease 2-like |
| NIATv7_g13385 | 13 | 22 | 12 | 38 | 43 | 53 | 26 | 21 | 28 | 30 | 44 | 32 | 10 | 10 | 10 | 31 | 24 | 20 | 13 | 16 | 15 | 22 | 17 | 15 | chalcone-flavanone isomerase-like protein |
| NIATv7_g15963 | 1 | 1 | 1 | 8 | 15 | 9 | 5 | 5 | 3 | 7 | 9 | 3 | 7 | 5 | 8 | 41 | 35 | 31 | 18 | 21 | 21 | 10 | 11 | 9 | phospholipase a1- chloroplastic-like |
| **NIATv7_g23173** | **8** | **7** | **10** | **20** | **37** | **21** | **12** | **13** | **10** | **16** | **21** | **11** | **6** | **7** | **6** | **19** | **17** | **14** | **10** | **11** | **8** | **12** | **11** | **8** | ***NtJAR*1.1** |
| NIATv7_g42489 | 2 | 2 | 2 | 37 | 45 | 39 | 24 | 22 | 27 | 33 | 38 | 26 | 25 | 16 | 22 | 88 | 83 | 82 | 40 | 64 | 51 | 28 | 34 | 26 | u-box domain-containing protein 21-like |

**Supplementary file 1C. Summary of RNA-seq reads from six closely related *Nicotiana* species.**

**Supplementary file 1D. Primers used for qPCR and VIGS in this study.**

| **Gene ID** | **Forward Primer** | **Reverse Primer** | **Experiment** |
| --- | --- | --- | --- |
| *NaLRRK1*(NIATv7_g00304) | CTAGATTTTCTTGATACCCTTTCTT | CCATCTTCGCGGTCATTACTTT | Open reading frame clone |
|  | TGTTGCTGGTTCTGTCGGTT | CCGGCCTTCTGGTGAGATAC | qPCR for silencing efficiency |
|  | GCGGCGGTCGACATATTCCTCCAGAATATGCA | GCGGCGGGATCCTAACACAAGCTAGAGCCAC | VIGS construct |
|  | CACCATGAACTTAAGGTATGCTC | CCTTGCATTGAGAAGCATGCGAA | Subcellular localization |
| *Plasma membrane intrinsic protein 2a* | CACCATGGCAAAGGATGTGGAAGC | GACGTTGGCAGCACTTCTGAA | Subcellular localization |
| *Elongation Factor 1α (D63396)* | CCACACTTCCCACATTGCTGT | CGCATGTCCCTCACAGCAAAAC | qPCR |
| *NaGLA* | AGTAGCAGATGATGTTAGTACATGTA | ACATGTGAATATGCCCATGGCATACT | qPCR |
| *NaLOX3* | GGCAGTGAAATTCAAAGTAAGAG | CCCAAAATTTGAATCCACAACA | qPCR |
| *NaAOS* | GACGGCAAGAGTTTTCCCAC | TAACCGCCGGTGAGTTCAGT | qPCR |
| *NaAOC* | CTATATACCGGAGACCTAAAGAAGA | AGTATCCTCGTAAGTCAAGTACGAT | qPCR |
| *NaCYP94B3-like1/2* | TGAAACGCGTTCACTGTTGT | GAGTGCCTTATCGCCATTGT | qPCR |
| *NaCYP94C1* | ACACTGTTGCTTCTGCGTTG | GTACCATCGGGGAGCGTAT | qPCR |
| *NaTPI* | TCAGGAGATAGTAAATATGGCTGTTCA | ATCTGCATGTTCCACATTGCTTA | qPCR |
| *NaTD* | ATTTTGCCTGTAACCCCAAA | GCCCCCTGATGGATTATTCT | qPCR |
| *NaMyb8* | AACCTCAAGAAACTCAGGACATACAA | GATGAATGTGTGACCAAATTTTCC | qPCR |
| *NIATv7_g00304* | AAATTCAACAAGGCCAAATCAG | TAAACTTCCATCAGTCTTTCCC | qPCR |
| *NIATv7_g10386* | TGTTGCTGGTTCTGTCGGTT | CCGGCCTTCTGGTGAGATAC | qPCR |
| *NIATv7_g21011* | TCAAATCATTGTTCTTGGCCTGG | GTTGTTTGAAGTCAAGTTAAGGACT | qPCR |
| *NIATv7_g26672* | TTTGGAGCTTCTTACAGGCAAA | CAGGTGGTGTGTTTATGTTGTC | qPCR |
| *NIATv7_g39903* | CCCTTCTTCCTCAACCACT | CATTAGGGAGGCTGCTGT | qPCR |
| *NIATv7_g42200* | CGGTGGATCTACTATTTTTCCT | TGAGAAGTGGGGCTATGTGC | qPCR |
| *NIATv7_g28967* | CGAGTCTTTTGGATTCGGTAA | ATGCTGTGACGCCATCATAC | qPCR |
| *NIATv7_g26266* | CAAAGTTGAGCAGAAACAGAACG | GCTTCCATGTTGGATTTGGAAGT | qPCR |
| *NIATv7_g08724* | AATTCCAAAATCAAGAGCAACCC | TTTCTGTTCGACTACTCTGCTCT | qPCR |
| *NIATv7_g15549* | GGTTCTTTGAATTGGGGTTG | GGGTAATGCAGTTTTTCCAAG | qPCR |
| *NIATv7_g17204* | GGAAGCGATTTGTCCTACTCTTC | AACAGCATTTCCACCAGAGAAAA | qPCR |
| *NIATv7_g05178* | GAGCGAATCGAAAACCAGTC | GGGTCTAATCCCGATAAAAGG | qPCR |
